# Antisynthetase Syndrome: The Classical Phenotype With a Twist

**DOI:** 10.7759/cureus.42360

**Published:** 2023-07-24

**Authors:** Achilleas Betsikos, Evanthia Gazouni, Spyridoula Bika, Eleni Paschou, Nikolaos Sabanis

**Affiliations:** 1 First Department of Internal Medicine, General Hospital of Trikala, Trikala, GRC; 2 Department of General Practice and Family Medicine, 10th Local Health Unit of Giannouli, Larisa, GRC; 3 Department of Nephrology, General Hospital of Trikala, Trikala, GRC

**Keywords:** anti-jo1 antibodies, inflammatory myopathies, myoglobinuric acute kidney injury, acute rhabdomyolysis, antisynhetase syndrome

## Abstract

Antisynthetase syndrome is a systemic autoimmune rheumatic disease characterized by multiple organ involvement, including interstitial lung disease, myositis, non-erosive arthritis, fever, Raynaud’s phenomenon, “mechanic’s hands,” and the presence of autoantibodies against aminoacyl-tRNA synthetases, mainly anti-Jo1 (histidyl) antibodies. Patients with antisynthetase syndrome and active muscle inflammation are usually presented with elevated creatine phosphokinase levels, even in the range of acute rhabdomyolysis. Despite that, the presence of myoglobinuric acute kidney injury is rarely seen in patients with myositis-associated rhabdomyolysis. Herein, we report the case of a 64-year-old man who presented with acute kidney injury due to severe rhabdomyolysis in the setting of antisynthetase syndrome diagnosed by the classical clinical triad of (1) interstitial lung disease, (2) non-erosive arthritis, and (3) active myositis and the presence of anti-Jo1 antibodies. The diagnosis was confirmed by muscle biopsy histological findings as well as electromyography. In this case report, we also discuss the classical clinical manifestations of antisynthetase syndrome and a twist toward this unusual complication associated with active muscle inflammation.

## Introduction

Antisynthetase syndrome refers to a rare, immune-mediated systematic disease. In its typical form, it encompasses the co-existence of myositis, interstitial lung disease, non-erosive arthritis, and the presence of autoantibodies against one of several aminoacyl-tRNA synthetases (ARS), most commonly anti-Jo1 (histidyl) antibodies. The classical phenotype also includes the presence of fever, Raynaud’s phenomenon, and skin lesions known as “mechanic’s hands” [1].

Most patients with antisynthetase syndrome and active muscle inflammation presented with elevated plasma creatine phosphokinase levels and several other skeletal muscle components released into the extracellular space, such as aldolase, lactate dehydrogenase (LDH), and aspartate aminotransferase (AST). Notably, in untreated patients with inflammatory myopathies, plasma creatine phosphokinase values tend to be up to tenfold greater than the upper limit of normal ones. The aforementioned levels are far greater than the limits employed to diagnose rhabdomyolysis in the literature [2].

Nevertheless, the occurrence of myoglobinuric acute renal failure induced by rhabdomyolysis is an exceptionally rare complication in the setting of idiopathic inflammatory myopathies. Hence, in this article, we aim to summarize the classical clinical manifestations of antisynthetase syndrome as well as the rarely seen complication of rhabdomyolysis-induced acute kidney injury as a result of severe muscle inflammation.

## Case presentation

A 64-year-old man was admitted to our hospital because of prolonged fever, muscle weakness, and fatigue. The patient presented to the emergency department reporting 15 days of fever, dark discoloration of the urine, progressively worsening fatigue, and muscle weakness mainly in the proximal muscles of the lower extremities. He appeared calm, alert, and oriented. On examination, the blood pressure was 106/50 mmHg, the heart rate 87 beats per minute, the temperature 36°C, and the oxygen saturation was 93% on ambient air. During aspiration, there were crackles at the bases of both lung fields. Supplemental oxygen was administered through a nasal cannula at a rate of 2 L/min. The patient was morbidly obese, with a body mass index of 40.3 kg/m^2^. When examining the musculature, a slight difficulty to rise from the seated position was noted, a finding congruent with proximal muscle weakness. The rest clinical examination was unremarkable.

Other medical histories included arterial hypertension, type 2 diabetes mellitus, hyperuricemia, and surgical history of thyroidectomy due to an underlying goiter. Three months ago, the patient was diagnosed SARS-Cov-2 positive with a mild course of the disease, not requiring hospitalization. His chronic medications included metformin, linagliptin, irbesartan, levothyroxine, and febuxostat, and no allergies were reported.

Blood and urine cultures were obtained, and a skin tuberculin test was performed. Treatment with ampicillin/sulbactam as well as a prophylactic dose of enoxaparin was initiated. Laboratory results were notable for exceedingly increased creatine phosphokinase (CPK) levels of 5605 mg/dL. It is also noteworthy that the results included leukocytosis, a moderate increase in kidney and liver function tests as well as increased D-dimer levels. Other results are shown in Table 1. Chest radiography revealed an increased cardiothoracic index. The nucleic acid test for SARS-Cov-2 was negative.

**Table 1:** Laboratory testing results on admission. SGOT: serum glutamic-oxaloacetic transaminase; SGPT: serum glutamate pyruvate transaminase; LDH: lactate dehydrogenase; CPK: creatine phosphokinase; ANCA MPO/PR3: antineutrophil cytoplasmic antibodies myeloperoxidase/anti-proteinase 3; anti-RNP: antinuclear ribonucleoprotein antibodies; ANA: antinuclear antibodies; anti-SSA: anti-Sjögren's syndrome-related antigen A autoantibodies; anti-SSB: anti-Sjögren's syndrome-related antigen B autoantibodies; anti-Scl-70: anti-sclerosis-70 antibodies; C3: complement factor 3; C4: complement factor 4.

Variables	On admission	Reference range
White blood cells (x10^3^/μL)	14.57	4-10.8
Hematocrit/hemoglobin (% /g/dL)	43.7/13.5	37.7-47.9/11.8-17.8
Platelets ( x10^3^/mL)	278	150-350
Total serum protein (g/dL)	6.28	6.4-8.3
Urea (mg/dL)	87	10-50
Creatinine (mg/dL)	2.51	0.40-1.10
Sodium (mmol/L)	135	136-145
Potassium (mmol/L)	5.5	3.5-5.1
SGOT (IU/L)	198	5-40
SGPT (IU/L)	92	10-37
LDH (IU/L)	502	135-225
CPK (IU/L)	5605	24-190
Calcium (mg/dL)	8.1	8.1-10.4
C-reactive protein (mg/dL)	6.36	<0.7
Procalcitonin	0.12	
Total bilirubin/direct bilirubin (mg/dL)	1.09/0.15	0.3-1.2/0-0.3
Fibrinogen (mg/dL)	444	200-400
International normalized ratio	1.03	
D-dimers (μg/mL)	3.63	0.00-0.25
Rheumatoid factor (U/mL)	7	0-14
Aldolase (U/L)	59.8	1.0-8.1
Anti-Jo1 (IU/mL)	276.9	Neg<10
ANCA MPO/PR3	Neg/Neg	
Anti-RNP (U/mL)	1.9	Neg<10
ANA/anti-SSA/SSB/anti-Scl-70	Neg/Neg/Neg	
C3 (g/L)	1.3	0.82-1.80
C4 (g/L)	0.28	0.10-0.40

**Figure 1:**
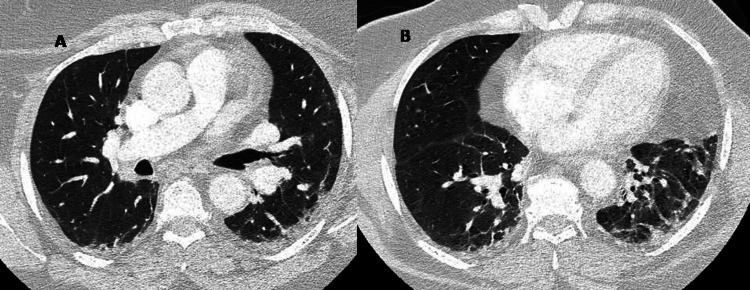
CT of the chest revealed (A) a non-specific interstitial pneumonia pattern characterized by diffuse ground glass opacities with associated reticular opacities and (B) lung architectural distortion. CT: computed tomography.

A fever of 38.2°C was recorded on the first day in the hospital and remained present until four days before discharge. During his hospitalization, the CPK elevation persisted and mild respiratory distress presenting as dyspnea developed. Chest computed tomography (CT) with intravenous contrast administration was negative for pulmonary embolism but revealed multiple bilateral ground glass opacities as well as lung architectural distortion that prompted urgent pulmonology evaluation for interstitial lung disease (Figure 1). Cardiology was also contacted to rule out cardiac causes of dyspnea; the echocardiography was unremarkable and the Troponin I test was negative. Examination of the hands showed diffuse bilateral arthritis. There was neither a rash nor lesions known as “mechanic’s hands.” Since urinalysis revealed tubular epithelial cells, granular casts, dark pigmented casts, and absence of red blood cells, the acute kidney injury on admission was attributed to rhabdomyolysis. After the administration of intravenous hydration with normal saline and alkalinization of urine, targeting a urinary pH greater than 6.5, along with the withdrawal of metformin and irbesartan, creatinine levels regressed gradually to lower levels. Tests for hepatitis A, B, and C, human immunodeficiency virus (HIV), cytomegalovirus, *Brucella* spp., and *Toxoplasma gondii* were negative. Skin tuberculin test, urine, sputum, and serial blood cultures were also negative. Immunological assays and aldolase blood levels were ordered.

**Figure 2:**
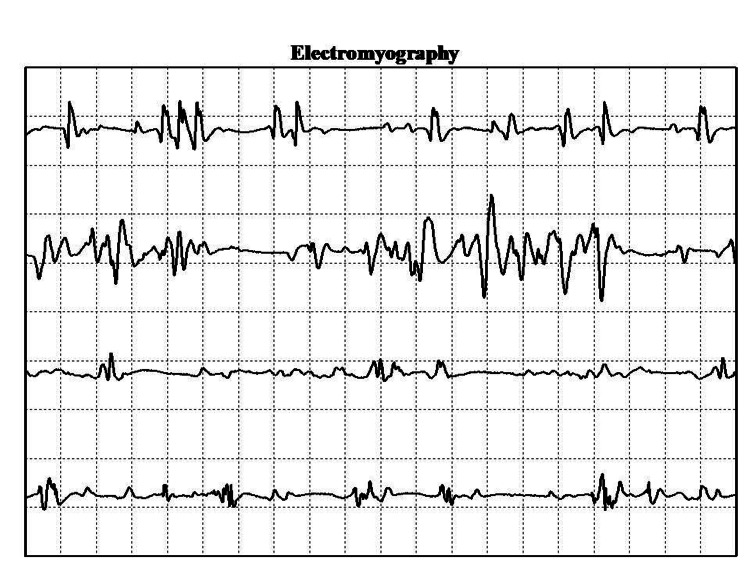
Electromyography showed characteristic myopathic lesions.

**Figure 3:**
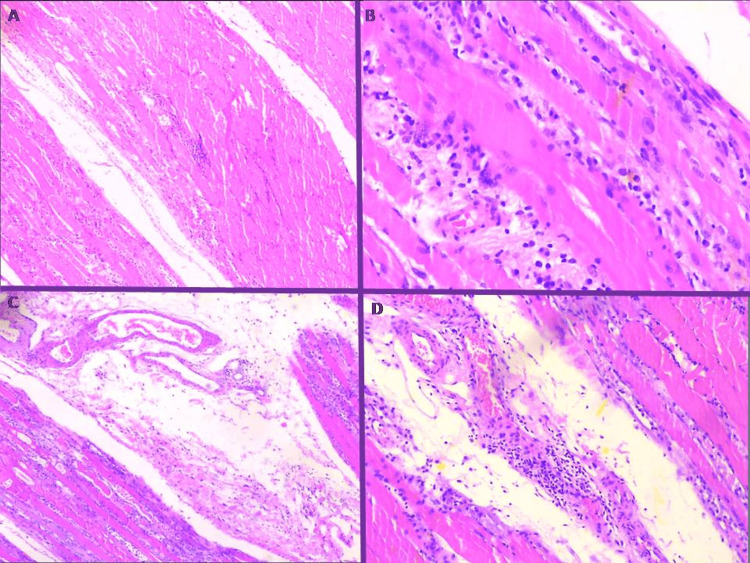
Muscle biopsy revealed characteristic extensive inflammatory infiltrates with lymphocytes and plasma cells (A, B) accompanied by perimysial pathology and perifascicular necrotizing myopathy (C), resulting in acidophilic homogenization alongside diffuse perivascular lymphocytic infiltrates of perimuscular fat (D).

On hospital day 5, the proximal muscle weakness had deteriorated to the point that the patient became bedridden. Inflammatory myositis was the proposed condition in the differential diagnosis. In order to distinguish between the different types, electromyography and muscle biopsy of the afflicted quadriceps were performed. The former test showed myopathic lesions, excluding neuromuscular disorders (Figure 2), while the latter test confirmed the diagnosis of inflammatory and necrotic myositis (Figure 3). Meanwhile, the immunological work-up was positive for anti-Jo1 antibodies with a titer of 276.9 IU/L (reference range <10 IU/L) and aldolase of 59.8 U/L (reference range 1-8.1 U/L).

Taken together, the coexistence of interstitial lung disease, inflammatory myositis in the presence of anti-Jo1 antibodies, and non-erosive arthritis constitutes the classical clinical triad of antisynthetase syndrome.

Treatment with methotrexate twice weekly, along with folic acid, vitamin D, and prophylactic dose of trimethoprim-sulfamethoxazole, was initiated. A three-day scheme of intravenous methylprednisolone pulse was administered empirically before the establishment of the diagnosis so that the progression of the lung disease could be prevented. Then, treatment was tapered with per os methylprednisolone. This patient’s fever resolved 24 hours after the initiation of the aforementioned treatment. The CPK levels were gradually normalized along with creatinine levels, and by the day the patient was discharged, there was no need for supplemental oxygen. The patient was referred to outpatient rheumatology follow-up and was advised to continue his physiotherapy to full recovery.

## Discussion

Antisynthetase syndrome represents a rare entity in the spectrum of inflammatory myositis. Its classic triad consists of myositis, interstitial lung disease, and arthritis, all of which were present in our case [3]. In the vast majority of cases, anti-Jo1 antibodies have a central role in the course of the disease and might even precede the clinical manifestations [4]. Other antisynthetase antibodies have been discovered, and a correlation with this syndrome has been established, albeit they have not yet been included in the diagnostic criteria of inflammatory myopathies [5,6].

Furthermore, this distinct syndrome might present with constitutional symptoms such as fever, Raynaud's phenomenon, sicca syndrome, skin lesions known as “mechanic’s hands,” or heliotropic-like rash that resembles dermatomyositis [7]. The severity of the disease varies broadly, with some people afflicted by a fulminant disease causing severe disability [8], while others present with mild proximal muscle weakness or even a subclinical course that remains undiagnosed. Of note, the syndrome might progress over time or relapse after treatment. Sometimes, there might be an overlapping of antisynthetase antibodies and other autoimmune disorder-specific antibodies such as systematic sclerosis and Sjogren’s syndrome [9].

More specifically, the presence of anti-SSA (Ro52) autoantibodies, a serological marker of Sjogren’s syndrome, has been correlated with a particular phenotype of antisynthetase syndrome, resulting in more severe myositis and joint involvement as well as an increased risk of cancer [10]. Our patient was not diagnosed with a combination of anti-Jo1 and anti-SSA/Ro antibodies that has also been associated with a severe, acute, and resistant to therapy form of lung disease [11].

In our case, this patient suffered an acute kidney injury due to rhabdomyolysis that resulted in myoglobinuria and granular casts. This complication of the syndrome is even rarer; only a handful of cases are described in the literature. With the breakdown of muscle, heavy chains of myoglobin and toxic cellular debris arising from the necrotic cells pose a direct threat to the kidney, such that aggressive intravenous hydration is warranted [12].

 Indeed, the histopathology injury pattern of our patient revealed severe necrotizing perifascicular myositis compatible with the one usually recognized in anti-Jo1 antibody-positive patients with myositis. The above specific morphological phenotype not only explains the significant elevated levels of CPK and the subsequent kidney injury but also differentiates antisynthetase syndrome from other idiopathic inflammatory myopathies, such as dermatomyositis, in which perifascicular atrophy is observed [13].

In support of the above, the study of the spectrum of renal involvement in a large cohort of patients with inflammatory myopathies revealed that the incidence of acute kidney injury is more frequent than previously thought. The main causes of acute kidney injury were determined as myoglobinuria-related acute tubular necrosis and drug-induced nephrotoxicity. In the same study, the researchers identified that male sex, diabetes mellitus, cardiovascular disease, and initial proteinuria >0.3 g/day were associated with the occurrence of acute kidney injury in these patients [14]. 

 Hence, this report emphasizes the need to keep in mind antisynthetase syndrome with active muscle inflammation as part of the differential diagnosis in rhabdomyolysis-associated acute kidney injury.

## Conclusions

Antisynthetase syndrome constitutes a rare immune-mediated clinical entity. Despite the shared features with the broader inflammatory spectrum of myositis, there are distinct clinical and immunological characteristics. Differentiation from other types of inflammatory myopathies is crucial. Once the diagnosis of antisynthetase syndrome is made, immediate administration of immunosuppressive treatment is required so that irreversible lung damage is prevented as well as other complications induced by active muscular inflammation, including myoglobinuric acute kidney injury.
